# Sutured Versus Sutureless Enterostomies: An Alternative Technique in Critically Sick Neonates

**DOI:** 10.7759/cureus.24057

**Published:** 2022-04-12

**Authors:** Ezza Ahmed, Nabila Talat, Hafiz M Adnan, Jamal Butt, Farrakh Mahmood Star, Anum Manzoor

**Affiliations:** 1 Pediatric Surgery, The Children's Hospital and University of Child Health Sciences, Lahore, PAK

**Keywords:** sick neonaates, pneumoperitoneum, necrotizing enterocolitis, meconium ileus, stoma related complications

## Abstract

Background and objective

Intestinal perforations and necrotizing enterocolitis (NEC) requiring the formation of temporary intestinal stoma are prevalent conditions worldwide. This prospective study aimed to address the following research question: does sutureless enterostomy lead to fewer complications compared to conventional enterostomy in critically sick surgical neonates who need a stoma?

Methods

We conducted a randomized control trial (TCTR20211011004) from October 2020 to October 2021. A total of 120 patients were randomized to the following two study groups: Group A: conventional sutured enterostomy; Group B: sutureless enterostomy, with 60 patients in each group. The operative time, complications, and mortality were compared between the two groups using the chi-squared test.

Results

The groups were comparable with respect to the mean age at presentation, gestational age, weight, and sex ratio. In Group A, the main operative diagnoses were NEC in 22 patients, pneumoperitoneum in 25, complicated meconium ileus in nine, bowel atresia in three, and midgut volvulus in one; while in Group B, 25 patients had NEC, 20 had pneumoperitoneum, eight had meconium ileus, two had bowel atresia, and five had midgut volvulus. The mean operative time (93.5 ± 28.71 minutes) was significantly longer in the conventional group compared to the sutureless group (52.08 ± 18.53 minutes). Oral feed was started significantly earlier in the sutureless stoma group. Mortality was 43.3% in Group A and 46.6% in Group B. Complication rate was significantly higher in sutured stoma group.

Conclusion

The management of critically sick neonates, especially those with NEC and intestinal perforation, still presents a surgical dilemma. Based on our findings, the sutureless enterostomy may reduce operative time and complications in critically sick neonates.

## Introduction

The term “ostomy” is a Greek word that means “mouth.” In surgery, enterostomy refers to a surgically fashioned intestinal opening [1]. Idiopathic intestinal perforations and necrotizing enterocolitis (NEC) are serious causes of mortality and morbidity in neonates. Spontaneous intestinal perforation (SIP) and NEC affect 3-6% and 7-8% of neonates respectively [2,3]. NEC also affects 10% of preterm infants [1]. The Japanese Society of Pediatric Surgeons stated that the mortality due to intestinal perforation ranged from 16.9% in 2003-2007 to 18.2% in 2008-2013, out of which 20% were due to NEC [4]. Emergency laparotomy and the formation of diversion enterostomy is the main strategy for treatment in critically sick, preterm, or extremely low-birth-weight (ELBW) neonates with intestinal perforation, NEC, and midgut volvulus. Durell et al. stated that 28% of neonates born at <26 weeks of gestation had to undergo emergency laparotomy and received a stoma [5]. Enterostomy-related complications are a significant cause of morbidity and mortality with a reported rate of 18-100% [5-8].

There are many techniques for the formation of enterostomies, namely double barrel ileostomy, loop ileostomy, exteriorization, and T-tube ileostomy; however, there is still a debate as to which technique is superior [8]. Phadke et al. described a sutureless technique of stoma formation in adults and introduced the concept of “delayed-primary self-maturation” (DPSM) [9]. Staple obstructed gut was exteriorized at the proposed site of stoma and, later when peristalsis was established, an opening was created by electrocautery at the antimesenteric border of the gut on the bedside. The mucosal cuff pouts out and fuses with the dermis. The use of staplers is unrealistic in neonates. Nose et al. have described a sutureless technique for the creation of enterostomies with cyanoacrylate adhesive in ELBW neonates [4]. Ohashi et al. published the treatment outcomes of 12 ELBW infants (ELBWIs) who were admitted to the neonatal intensive care unit (NICU) over a seven-year period from 2007 to 2013 and had sutureless enterostomy with near-zero early and late complications [7]. However, there are very few reports that offer a comparison between different techniques of stoma formation in the published literature.

In our setting, we deal with critically sick neonates who require diversion enterostomies very often and the idea of DPSM of the stoma can be a potential game-changer for them as it reduces the operative time and causes low complication rates. The aim of this study is to fill the gap in the previously published literature by comparing the two techniques of stoma formation and to test the hypothesis that sutureless enterostomies lead to near-zero complication rates in early and late postoperative periods as compared to sutured stomas in critically sick neonates.

## Materials and methods

A randomized controlled trial was conducted after obtaining approval from the Institutional Ethical Committee of the Children’s Hospital and University of Child Health Sciences, Lahore, Pakistan (letter number: CHICH/2021-216) from October 2020 to October 2021 in the Department of Pediatric Surgery. The trial was registered in the Thai Clinical Trial Registry (TCTR202110110040). A total of 120 patients were included in this study and were equally randomized by non-probability purposive sampling method into two groups of 60 patients each: Group A: conventional enterostomy, and Group B: sutureless enterostomy. Informed and written procedure-specific consents were sought from parents or guardians of all the study patients. Sick, preterm, and LBW neonates who required emergency laparotomy and enterostomy due to pneumoperitoneum or NEC were included. Cases of elective enterostomies due to anorectal malformation and Hirschsprung’s disease were excluded. Demographics, birth weight, disease process, stoma type, hospital stay, the requirement of total parenteral nutrition (TPN), and early and late complications were recorded.

All patients were resuscitated with intravenous fluids and antibiotics and were optimized for laparotomy. Input and output were monitored. All baseline investigations were done. High-risk and procedure-specific informed consent was taken. Patients were equally randomized by the non-probability purposive sampling method to Group A and Group B. In Group A, sutured stoma was developed. Stoma was anchored with muscle and fixed on the skin; while in Group B, sutureless stomas were formed. A small supraumbilical transverse laparotomy incision was made. The gut was exteriorized from a site away from the wound, and muscles of laparotomy were closed using absorbable suture followed by skin. Stoma was not anchored with the muscles or matured. Adjustments to the wound were made while performing this procedure to avoid the obstruction of the intestinal lumen.

Stoma was evaluated and examined for necrosis, bleeding, and perforation till the discharge of the patient after primary surgery. The patients were kept in the surgical NICU and were discharged once the enteral feed was established and biochemical parameters were normal.

A follow-up was done weekly for four weeks in the stoma clinic, and patients were evaluated for late stoma-related complications including stenosis, retraction, prolapse of stoma, and excoriation of the skin. The data analysis was performed using SPSS Statistics version 26.0 (IBM, Armonk, NY). Mean and standard deviation (SD) were calculated for quantitative variables. The chi-squared test was used, and a p-value ≤0.05 was considered statistically significant. Frequencies and percentages were calculated for qualitative variables.

## Results

A total of 120 infants underwent laparotomy and the creation of intestinal stomas during the study period. Out of those, 60 infants were treated using a novel sutureless technique. The mean age, weight, and gestational age in Group A and Group B were comparable, while the female-to-male ratio was 1:1.1 and 1:1.2 respectively; 20 (33.1%) patients in Group A having conventional enterostomies had early stoma-related complications, while complications in Group B were found in seven (11.6%) patients. On statistical analysis, the difference in early stoma-related complications in both groups was significant (p=0.021) (Table1). The most common late complication was peristomal excoriation in the conventional group, probably due to the anchorage of the stoma on the skin. On statistical analysis, the difference in late stoma-related complications was significant (p=0.036) (Table 1).

**Table 1:** Mean values of statistical variables for Groups A and B *Mean ± standard deviation. **Among survived neonates after four weeks of surgery

Variables	Group A (n=60)	Group B (n=60)	P-value
Age (days)	9.40 ± 7.069*	10.38 ± 8.30*	0.45
Weight (kg)	1.88 ± 0.53*	1.86 ± 0.55*	0.73
Gestational age (weeks)	34.7 ± 2.59*	34.7 ± 2.92*	0.68
Establishment of enteral feed (days)	5.2 ± 1.48*	3.6 ± 1.00*	0.001
Mean operative time (minutes)	93.5 ± 28.71*	52.08 ± 18.53*	0.03
Early complications	20 (33.1%)	7 (11.6%)	0.021
Bleeding	5 (8.3%)	7 (11.6%)	
Necrosis	7 (11.6%)	------	
Peristomal fistula	4 (6.66%)	------	
Mucocutaneous disruption	4 (6.66%)	-------	
Late complications**	18 (52.9%)	8 (25%)	0.036
Stenosis	3 (8.8%)	1 (3.1%)	
Peristomal excoriation	11 (32.33%)	6 (18.7%)	
Peristomal herniation	1 (2.9%)	------	
Prolapse	3 (8.8%)	1 (3.1%)	
Hospital stay (days)	7.11 ± 1.45*	5.84 ± 1.46*	0.003
Outcome	Survived=34	Survived=32	0.427
	Mortality=26	Mortality=28	

Underlying illnesses in Group A were as follows: NEC in 22 (36.6%) patients, pneumoperitoneum (due to NEC, Hirschsprung's disease, and meconium ileus) in 25 (41.6%), meconium ileus in nine (15%), small bowel atresia in three (5%), and midgut volvulus in one (1.6%) patient. In Group B with sutureless enterostomies, 25 (41.6%) patients had NEC, 20 (33.3%) patients had pneumoperitoneum, eight (13.3%) of the patients had meconium ileus, two (3.33%) suffered from small bowel atresia, and five (8.33%) of the patients had volvulus (Table 2).

**Table 2:** Diagnosis of patients in Group A (sutured enterostomies) and Group B (sutureless enterostomies) NEC: necrotizing enterocolitis; HD: Hirschsprung's disease

Diagnosis	Groups, n (%)		Total
	Sutured (Group A)	Sutureless (Group B)	
NEC	22 (36.6%)	25 (41.6%)	47
Pneumoperitoneum due to NEC	19 (31.6%)	16 (26.6%)	35
Pneumoperitoneum due to HD	3 (5%)	2 (3.3%)	5
Pneumoperitoneum due to meconium ileus	3 (5%)	2 (3.3%)	4
Meconium ileus	9 (15%)	8 (13.3%)	17
Bowel atresia	3 (5%)	2 (3.3%)	5
Midgut volvulus	1 (1.6%)	5 (8.3%)	6
Total	60	60	120

**Figure 1:**
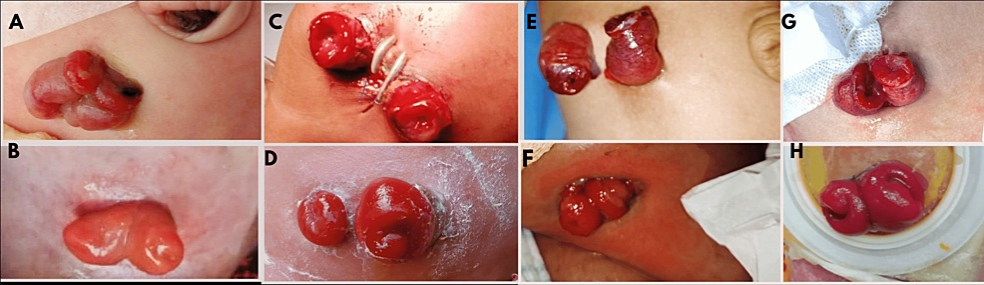
Immediate postoperative pictures of sutureless enterostomies (A, C, E, G), and images at follow-up after four weeks (B, D, F, H)

The mortality rate was 43.3% in Group A and 46.6% in Group B, and it was related to sepsis, idiopathic thrombocytopenia, multiorgan failure, and acute respiratory distress syndrome (ARDS). Immediate postoperative and follow-up pictures of sutureless enterostomies are shown in Figure 1.

## Discussion

Better healthcare facilities have improved the survival rate among sick and ELBW neonates in recent years. This has led to an increase in the number of enterostomies performed in developed and developing countries alike. In every setup, early and late complications related to stoma are common, and studies have been done aiming to map out the core issues leading to complications. Ohashi et al. suggested ischemia due to suturing and exterior compression to be the cause of early complications [7] and advocated the concept of DPSM, which was coined by Phadke et al. [9]. With recent advances and the prevalence of abbreviated laparotomy and damage-control surgeries, different techniques of stoma formation in neonates have evolved. Manterola et al. introduced the concept of floating stoma for the control of septic focus followed by delayed maturation of stoma [10]. Kondo et al. conducted a study on 21 neonates and introduced the stitching of stoma with a strip of gauze as damage-control surgery [11]. Nose et al. reported a new sutureless technique using cyanoacrylate on eight ELBW neonates, and no early or late stoma-related complication was found [4].

Simon et al. conducted a study on 30 ELBW neonates undergoing ileostomy at the Kepler University Hospital in Linz, Austria. The median gestational age, weight, and patient age were 25 ± 5 weeks, 887 g, and 18 days respectively, while in our conventional group (Group A), the mean gestational age was 34.7 ± 2.59 weeks, the mean weight was 1.88 ± 0.53 kg, and the mean age at presentation was 9.40 ± 7.069 days. The mean age and weight in both of our groups were comparatively higher than those in the previous studies as the survival rates among ELBW and premature neonates are very low in our country.

Ohashi et al. conducted a study on 12 ELBW patients by using the novel sutureless technique. The mean age at presentation was 19 ± 16 days, the mean weight was 671 ± 158 g, and the mean duration of surgery was 75 ± 35 minutes [7]. In our Group B patients (sutureless technique), the mean age at presentation was 10.38 ± 8.30 days, the mean weight was 1.86 ± 0.55 kg, and the duration of the procedure was 52.08 ± 18.53 minutes. The duration of the procedure was shorter as compared to Group A as well as that reported in previous literature. Short anesthesia duration results in a short procedure time, which reduces the neurotoxic effects on neonates [12]. The mean establishment time for full enteral feed in the conventional stoma group was 5.2 days and that in the sutureless stoma group was 3.6 days. According to a previous study, the enteral feed was established on the eighth postoperative day in NEC patients and the sixth postoperative day in patients with intestinal perforation in conventional stoma [13]. Short parenteral nutrition is always a stated goal in developing countries with limited resources as the main cause of mortality in Pakistan is sepsis.

Wolf et al. conducted a study on 76 neonates, and enterostomy-related complication was found in 82% of patients [14]. Stoma-related complications are higher in sick and ELBW neonates, especially those with NEC, as NEC is characterized by intestinal ischemia and generalized hypoxia [2]. In our study, we have compared two enterostomy formation techniques and their complications. Bælum et al. conducted a study on neonates with NEC, and stoma-related complications were found in 71% of patients, the most common being stoma stenosis in 68% of patients [3], whereas, in our conventional group, stoma-related early and late complications were found in 33.1% and 52.9% of patients respectively. The most common early complication was stoma necrosis with the median day of appearance being the third postoperative day, and the most common late complication was peristomal excoriation. In our sutureless group, 11% of patients had stoma-related early complications, mainly bleeding from the stoma, and the most common late complication was skin excoriation (25%). None of the patients had stoma necrosis. Stoma necrosis in sutured stoma occurs mainly due to blood-supply compromise. The thickness of the intestinal wall in neonates is similar to the diameter of the needles of sutures commonly used, which is 70 μm for 6-0, and 100 μm for 5-0, resulting in chances for bowel perforation and peristomal fistula. Peristomal fistula was noted in four patients in the conventional group, and patients had to undergo re-exploration. None of the patients in the sutureless group had this complication.

Mortality in our conventional group (Group A) was found to be 43%, and it was 46.6% in the sutureless group (Group B). The reported postoperative mortality in NEC was 35-50%, while Carmen et al. have published a retrospective review on the management of ELBW neonates with NEC in which spontaneous intestinal perforation and mortality was 18% [5-8,13]. In the study group of Ohashi et al., the mortality was 30% [7]. Mortality in both groups was related to sepsis, multiorgan dysfunction, resistance to antibiotics, hospital-acquired pneumonia, and ARDS. None of the mortality in both groups was related to enterostomy techniques.

Neonatal surgery and neonatal postoperative care are still a challenge in underdeveloped and developing countries including Pakistan. This study involves the largest number of patients who underwent the sutureless technique in the published literature. Although the mortality rate was high in the sutureless group, which is not related to enterostomy techniques, the absence of stoma necrosis and peristomal fistula in sutureless patients seems promising.

This study has a few limitations. Firstly, it was a single-center analysis. Secondly, it was conducted among critically sick neonates with comorbidities.

## Conclusions

The management of critically sick neonates with NEC and intestinal perforation still presents a surgical dilemma. Based on our comparison of a large number of patients with conventional and sutureless techniques, we can conclude that the patients who underwent the sutureless technique presented fewer complications, including the absence of stoma necrosis and peristomal fistula as well as a reduced duration of the procedure. These advantages offer a strong case for the adoption of the sutureless technique over conventional enterostomy.

## References

[1] Ambe PC, Kurz NR, Nitschke C, Odeh SF, Möslein G, Zirngibl H (2018). Intestinal ostomy. Dtsch Arztebl Int.

[2] Alganabi M, Lee C, Bindi E, Li B, Pierro A (2019). Recent advances in understanding necrotizing enterocolitis. F1000Res.

[3] Bælum JK, Rasmussen L, Qvist N, Ellebæk MB (2019). Enterostomy complications in necrotizing enterocolitis (NEC) surgery, a retrospective chart review at Odense University Hospital. BMC Pediatr.

[4] Nose S, Sasaki T, Saka R, Minagawa K, Okuyama H (2016). A sutureless technique using cyanoacrylate adhesives when creating a stoma for extremely low birth weight infants. Springerplus.

[5] Coletta R, Zulli A, O'Shea K, Mussi E, Bianchi A, Morabito A (2022). Minimizing enterostomy complication in neonates, lessons learnt from three European tertiary centres. Children (Basel).

[6] de Haro Jorge I, Prat Ortells J, Albert Cazalla A, Muñoz Fernández E, Castañón García-Alix M (2016). Long term outcome of preterm infants with isolated intestinal perforation: a comparison between primary anastomosis and ileostomy. J Pediatr Surg.

[7] Ohashi K, Koshinaga T, Uehara S, Furuya T, Kaneda H, Kawashima H, Ikeda T (2017). Sutureless enterostomy for extremely low birth weight infants. J Pediatr Surg.

[8] Kargl S, Wagner O, Pumberger W (2017). Ileostomy complications in infants less than 1500 grams - frequent but manageable. J Neonatal Surg.

[9] Phadke MV, Stocks LH, Phadke YG (2007). New "sutureless" technique of ileostomy and colostomy. Surg Endosc.

[10] Manterola C, Flores P, Otzen T (2016). Floating stoma: an alternative strategy in the context of damage control surgery. J Visc Surg.

[11] Kondo K, Chijiiwa K, Mukai M (2008). New technique for enterostomy of extremely low-birth-weight infants--intestinal anchoring with gauze. J Pediatr Surg.

[12] Marlow N (2014). Anesthesia and long-term outcomes after neonatal intensive care. Paediatr Anaesth.

[13] Eicher C, Seitz G, Bevot A (2012). Surgical management of extremely low birth weight infants with neonatal bowel perforation: a single-center experience and a review of the literature. Neonatology.

[14] Wolf L, Gfroerer S, Fiegel H, Rolle U (2018). Complications of newborn enterostomies. World J Clin Cases.

